# A risk prediction model for postoperative recovery of closed calcaneal fracture: a retrospective study

**DOI:** 10.1186/s13018-023-04087-8

**Published:** 2023-08-22

**Authors:** Wenjing Li, Yan Wang, Zenglei Zhang, Wei Chen, Hongzhi Lv, Yingze Zhang

**Affiliations:** 1 Hebei Provincial Key Laboratory of Orthopaedic Biomechanics, Hebei Orthopaedic Research Institute, No. 139 Ziqiang Road, Shijiazhuang, 050051 China; 2 Trauma Emergency Center, The Third Hospital of Hebei Medical University, No. 139 Ziqiang Road, Shijiazhuang, 050051 China; 3 Rehabilitation Center, The Third Hospital of Hebei Medical University, No. 139 Ziqiang Road, Shijiazhuang, 050051 China

**Keywords:** Calcaneal fracture, Postoperative recovery, Risk factors, Nomogram

## Abstract

**Objective:**

To explore the risk factors for postoperative recovery of closed calcaneal fracture and develop a prediction model.

**Methods:**

We retrospectively enrolled patients with closed calcaneal fracture from January 1, 2017 to December 31, 2020. Patients treated from 2017 to 2019 were selected as a training cohort and those in 2020 as a validation cohort. The outcome variable was the postoperative recovery evaluated by the Creighton-Nebraska calcaneal fracture scoring system. Multivariate logistic regression analysis was used to screen the risk factors of postoperative recovery. A risk prediction model was constructed in the training cohort and the corresponding nomogram was drawn. The model was validated internally using bootstrapping and externally by calculating the performance in the validation cohort.

**Results:**

A total of 659 patients with closed calcaneal fracture met the inclusion and exclusion criteria, which were divided into the training cohort (n = 509) and the validation cohort (n = 150). 540 cases (81.9%) patients recovered well after calcaneal fracture surgery. According to multivariate logistic regression analysis, female (*OR* = 2.525, 95% *CI* 1.283–4.969), > 60 years (*OR* = 6.644, 95% *CI* 1.243–35.522), surgery within 8–14 days after fracture (*OR* = 2.172, 95% *CI* 1.259–3.745), postoperative infection (*OR* = 4.613, 95% *CI* 1.382–15.393), and weight-bearing time longer than 3 months after surgery (4–6 months, *OR* = 2.885, 95% *CI* 1.696–4.907; 7–12 months, *OR* = 3.030, 95% *CI* 1.212–7.578; > 12 months, *OR* = 15.589, 95% *CI* 3.244–74.912) were independent risk factors for postoperative recovery of calcaneal fractures. The C-indices were 0.750(95% *CI* 0.692–0.808) in the training cohort and 0.688(95% *CI* 0.571–0.804) in the external validation cohort, and the C-index of internal validation was 0.715. The Hosmer–Lemeshow test showed good fitting of the model (all* P* > 0.05), which was consistent with the results of the calibration plots. Decision Curve Analysis indicated that the clinical effectiveness was the best when the threshold probability was between 0.10 and 0.45.

**Conclusions:**

Patients with female, > 60 years, surgery within 8–14 days after fracture, postoperative infection, and weight-bearing time longer than 3 months after surgery are more likely to have poor postoperative recovery. The risk prediction of fracture patients through this model might be translated into clinical guidance and application.

*Trial registration* This study was registered on the Chinese Clinical Trial Registry (Registration number: ChiCTR-EPR-15005878).

## Introduction

The calcaneus, located at the rear of the foot, is the main bony structure of the human body’s weight bearing. Calcaneal fractures, accounting for 30.35% of foot fractures [1], are caused by high-energy injuries such as falling from a height or traffic accidents, and generally occur in young and middle-aged male patients. At present, treatments of calcaneal fracture generally involve open or closed reduction and internal fixation; although it is superior to non-operative treatment [2], this surgery is complicated by the poor recovery of ankle function after operation. Malunion of the calcaneal body, poor wound healing, skin flap necrosis and other problems remain to be solved [3, 4]. Some scholars have studied the influencing factors of postoperative functional recovery, hoping to find a solution. Using its influencing factors to develop a risk prediction model is of great significance to judge the prognosis of patients with calcaneal fracture, and to take preventive measures.

Risk prediction model is a multi-factor model to predict the probability of suffering from a disease or a future outcome, which has great application value to the prevention and treatment of clinical diseases [5, 6]. There are many factors affecting the postoperative functional recovery of calcaneal fracture, such as injury mechanism, fracture type and wound location [7], which have been the focus of many researchers. In terms of prediction models, there are many studies on postoperative prediction models of femoral fracture and osteosarcoma [8, 9], but there is a lack of models related to the postoperative curative effect of calcaneal fracture.

Therefore, we designed this study to retrospectively collect the data of patients with closed calcaneal fracture admitted to our hospital from January 2017 to December 2020. The characteristics of preoperative, intraoperative and postoperative factors were described and analyzed. Our objective was to identify independent risk factors affecting the fracture recovery, and develop a risk predictive model for recovery of closed calcaneal fracture, using it to predict the high-risk groups with poor recovery, with the aim of guiding the postoperative nursing of patients and avoiding adverse situations.

## Methods

### Patient selection

This study collected the clinical data of patients with calcaneal fracture treated in our hospital from January 1, 2017 to December 31, 2020, and was approved by the Ethics Committee of our Hospital (Sect. 2015–002-1). This retrospective study was based on historical medical records and imaging data, and written informed consent was obtained from each participant prior to data collection. The inclusion criteria were as follows: (i)closed calcaneal fracture, (ii) open or closed internal fixation, (iii) follow-up for more than 12 months, (iv) complete medical records and imaging data, and (v) new and traumatic fracture. The exclusion criteria were as follows: (i)open calcaneal fracture, (ii) old calcaneal fracture or secondary fracture, (iii) pathological calcaneal fracture, (iv) manual reduction, fusion, lesion resection etc., (v) loss to follow-up or follow up for less than 12 months, and (vi) unclear diagnosis or incomplete data. New fracture refers to the fracture occurring within 3 weeks. And old fracture refers to the fracture occurring for 3 weeks or more, which is not treated in time and usually develops into malunion, delayed union or non-union [10]. Pathological fracture refers to the fracture caused by diseases resulting in the destruction of bone tissue, bone changes, and the reduction of bone biomechanical strength [11]. Traumatic fracture is the fracture caused by direct or indirect violent trauma [12]. Patients admitted from January 2017 to December 2019 were selected as the training cohort, and patients admitted from January to December 2020 were selected as the validation cohort.

### Data collection

Through telephone follow-up and medical record inquiry, the following research contents were collected: (i) preoperative factors: Gender, Age, Ethnic origin, Occupation, Body mass index (BMI), Season, Sanders classification, Injury cause, Preoperative combined injuries, Preoperative complications(Diabetes, Hypertension, Coronary heart disease, Respiratory system disease, and others), and Preoperative blister; (ii) intraoperative factors: Waiting time for surgery, Operation method, Incision selection, Internal fixation, and Anesthesia; (iii) postoperative factors: Postoperative infection, Deep vein thrombosis of lower extremities, Reduction quality, Rehabilitation training, and Weight-bearing time.

Patients were divided into six groups by age: 0–20 years old, 21–30 years old, 31–40 years old, 41–50 years old, 51–60 years old and > 60 years old. The predominant type of fracture was Sanders classification, supplemented by Essex-Lopresti and AO/OTA classification. The incisions were selected as small incision [13], S-shaped incision, L-shaped incision and 八-shaped incision. Reduction standard of calcaneal fracture: anatomical reduction or close to anatomic reduction, articular surface displacement ≤ 3 mm; restoration of overall calcaneal shape and geometric parameters of length, width and height; restoration of Gissane angle and Bohler's angle [14]. Judgments were made based on the imaging data of patients reviewed before surgery and one year after surgery.

### Outcomes

The Creighton-Nebraska calcaneal fracture scoring system evaluates the curative effect of calcaneal fracture [15]. It is the most widely used calcaneal fracture scoring system worldwide, evaluating pain, activity, range of activity, returning to work, changing shoe size and swelling. The highest score is 100, with 90–100 being excellent, 80–89 good, 65–79 fair, and < 65 as poor. In this study, the Creighton-Nebraska score was divided into a binary variable, with 80–100 as the good group and < 80 as the poor group. We also used the Rowe calcaneal fracture scoring system to assist in evaluation, including pain, range of activity, gait, activity, working condition.

### Statistical analysis

Statistical analyses were performed using R4.3.0 statistical software (R Foundation for Statistical Computing, Austria). All the factors collected were categorical variables, which were statistically described by frequencies and proportions. The comparison between groups was conducted by *χ*^*2*^ test or Fisher’s exact test. In the training cohort, the collected variables were analyzed by univariate analysis; variables with *P* < 0.20 were included in the multivariate logistic analysis, and the independent risk factors related to the postoperative curative effect of calcaneal fracture were obtained. Statistical significance was set at *P* < 0.05.

Variables selected by multivariate analysis were used as the final predictors to establish a risk prediction model for the postoperative recovery of calcaneal fracture, presented as a nomogram. The model validation can be divided into three parts: discrimination, calibration, and clinical effectiveness. The C-index is the main index to evaluate the discrimination of the model, as is the same as the area under the receiver operating characteristic (ROC) curve in the multivariate logistic regression model. The value ranges from 0.50 to 1.00, which is bounded by 0.70 and 0.90, corresponding to low, medium, and high discriminations, respectively [16]. The Hosmer–Lemeshow test (H–L test) was used to test the calibration of the model. A* P* > 0.05 indicates a strong goodness of fit between the predicted value and the actual value of the model, and a high calibration. As a visual form of calibration, in the calibration plot, the closer the actual prediction curve is to the ideal curve, the higher the calibration [17, 18].

We performed an internal validation using the C-index and calibration plot by bootstrap resampling in the training cohort. The external validity of the model was determined in the validation cohort by computing the C-indices, calibration plots, and H–L goodness of fit test. The clinical effectiveness was evaluated in the training and validation cohort using the decision curve analysis (DCA) curve, and the net clinical benefit of the model was obtained [19].

## Results

### Study populations

**Fig. 1:**
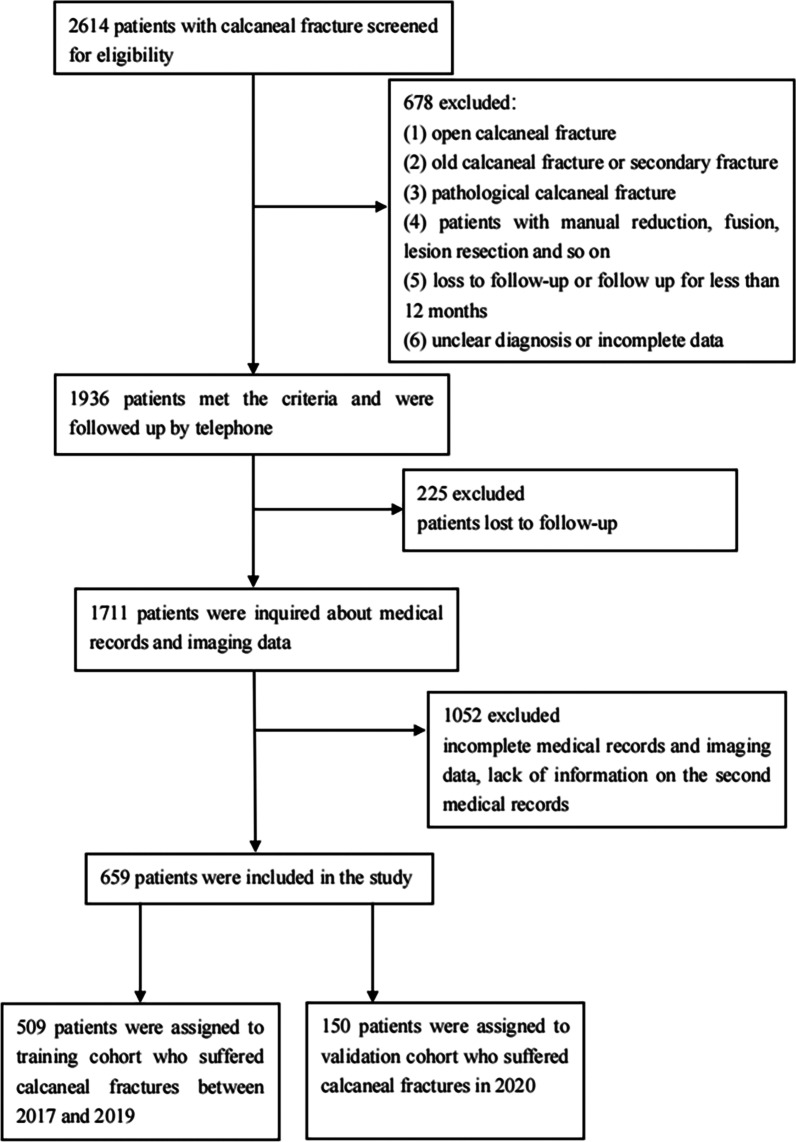
The screening process of research objects

**Fig. 2:**
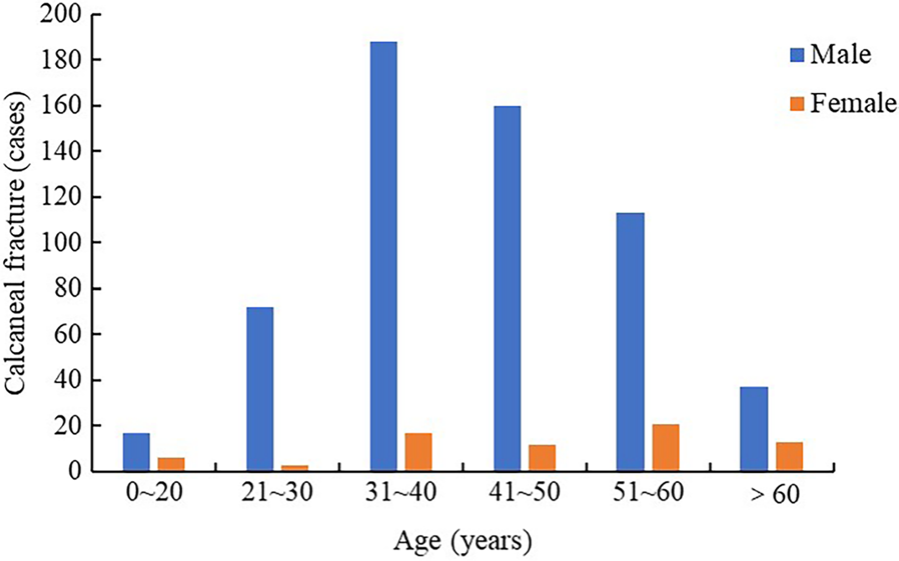
Gender and age distribution of patients with calcaneal fracture

A total of 659 patients with calcaneal fracture were enrolled in this study, and followed up for 12–48 months (mean 31.8): 509 patients were assigned to the training cohort and 150 patients to the validation cohort (Fig. 1). The Creighton-Nebraska score was obtained in the last follow-up: 540 cases (81.9%) obtained good scores, while 524 cases (79.5%) had Rowe scores above 80. The mean ± SD age was 42.7 ± 12.1 years old, and there were 587 males (89.1%) and 72 females (10.9%), with a male-to-female ratio of 8.2:1. As shown in Fig. 2, the number of calcaneal fracture patients under 30 years old showed an upward trend with increasing age, while the number of calcaneal fracture patients after 30 years old gradually decreased, and male patients accounted for a large proportion in all age groups. Farmer was the most common occupation (45.5%, 300 cases). In terms of BMI, overweight patients predominated (44.2%, 291 cases). Most patients were hospitalized in autumn (33.7%, 222 cases). Among the types of fractures, Sanders II accounted for 39.6%. Type II (98.0%) and Type C (98.0%) were the highest for the Essex-Lopresti and AO/OTA classification, respectively. Among the causes of injury, high-altitude falls was the main cause of fracture (93.5%). Fracture patients without preoperative combined injuries accounted for 65.4% (431 cases), and among patients, 81 had hypertension before surgery, 32 had diabetes, 30 had respiratory system disease, and 29 had coronary heart disease. Most patients (61.8%, 407 cases) underwent surgery within one week after injury and were treated with open reduction and internal fixation (85.9%, 566 cases), plate and screw (63.7%, 420 cases), small incisions (44.9%, 296 cases), and general anesthesia (54.9%, 362 cases). Most of the patients did not receive professional rehabilitation training (97.6%, 643 cases), and 62 patients had postoperative complications. Few patients (24.6%) did not meet the reduction criteria. The patients with postoperative weight-bearing time of 0–3 months accounted for a relatively large proportion (60.4%, 398 cases). A comparison of the baseline data between the training cohort and the validation cohort is shown in Table 1.

**Table 1:** Characteristics of calcaneal fracture patients [n (%)]

Variable	All	Training cohort	Validation cohort	*χ*^*2*^ value	*P* value
Calcaneus score				0.049	0.825
Good	540 (81.9)	418 (82.1)	122 (81.3)		
Poor	119 (18.1)	91 (17.9)	28 (18.7)		
Gender				0.230	0.631
Male	587 (89.1)	455 (89.4)	132 (88.0)		
Female	72 (10.9)	54 (10.6)	18 (12.0)		
Age (years)				1.258	0.939
0–20	23 (3.5)	19 (3.7)	4 (2.7)		
21–30	75 (11.4)	58 (11.4)	17 (11.3)		
31–40	205 (31.1)	158 (31.0)	47 (31.3)		
41–50	172 (26.1)	133 (26.1)	39 (26.0)		
51–60	134 (20.3)	105 (20.6)	29 (19.3)		
> 60	50 (7.6)	36 (7.1)	14 (9.3)		
Ethnic origin				1.669	0.196
Han	628 (95.3)	488 (95.9)	140 (93.3)		
Others	31 (4.7)	21 (4.1)	10 (6.7)		
Occupation				3.077	0.688
Student	20 (3.0)	16 (3.1)	4 (2.7)		
Office worker	58 (8.8)	41 (8.1)	17 (11.3)		
Farmer	300 (45.5)	229 (45.0)	71 (47.3)		
Manual worker	82 (12.4)	64 (12.6)	18 (12.0)		
Retired or Unemployed	27 (4.1)	20 (3.9)	7 (4.7)		
Others	172 (26.1)	139 (27.3)	33 (22.0)		
BMI (kg/m^2^)				2.792	0.425
< 18.5	17 (2.6)	14 (2.8)	3 (2.0)		
18.5–23.9	243 (36.9)	194 (38.1)	49 (32.7)		
24–27.9	291 (44.2)	223 (43.8)	68 (45.3)		
≥ 28.0	108 (16.4)	78 (15.3)	30 (20.0)		
Season				6.063	0.109
Spring	155 (23.5)	122 (24.0)	33 (22.0)		
Summer	203 (30.8)	167 (32.8)	36 (24.0)		
Autumn	222 (33.7)	162 (31.8)	60 (40.0)		
Winter	79 (12.0)	58 (11.4)	21 (14.0)		
Sanders classification				1.560	0.668
I	39 (5.9)	33 (6.5)	6 (4.0)		
II	261 (39.6)	198 (38.9)	63 (42.0)		
III	253 (38.4)	195 (38.3)	58 (38.7)		
IV	106 (16.1)	83 (16.3)	23 (15.3)		
Injury cause				5.642	0.130
Traffic accident	18 (2.7)	13 (2.6)	5 (3.3)		
Fall on the flat ground	12 (1.8)	11 (2.2)	1 (0.7)		
Fall from a high altitude	616 (93.5)	472 (92.7)	144 (96.0)		
Other	13 (2.0)	13 (2.6)	0 (0.0)		
Preoperative combined injuries				2.505	0.114
Yes	228 (34.6)	168 (33.0)	60 (40.0)		
No	431 (65.4)	341 (67.0)	90 (60.0)		
Diabetes				0.015	0.902
Yes	32 (4.9)	25 (4.9)	7 (4.7)		
No	627 (95.1)	484 (95.1)	143 (95.3)		
Hypertension				0.196	0.658
Yes	81 (12.3)	61 (12.0)	20 (13.3)		
No	578 (87.7)	448 (88.0)	130 (86.7)		
Coronary heart disease				2.370	0.124
Yes	29 (4.4)	19 (3.7)	10 (6.7)		
No	630 (95.6)	490 (96.3)	140 (93.3)		
Respiratory system disease				0.937	0.333
Yes	30 (4.6)	21 (4.1)	9 (6.0)		
No	629 (95.4)	488 (95.9)	141 (94.0)		
Other preoperative complications				0.007	0.933
Yes	56 (8.5)	43 (8.4)	13 (8.7)		
No	603 (91.5)	466 (91.6)	137 (91.3)		
Preoperative blister				1.283	0.257
Yes	39 (5.9)	33 (6.5)	6 (4.0)		
No	620 (94.1)	476 (93.5)	144 (96.0)		
Waiting time for surgery (days)				0.532	0.766
0–7	407 (61.8)	318 (62.5)	89 (59.3)		
8–14	200 (30.3)	151 (29.7)	49 (32.7)		
> 14	52 (7.9)	40 (7.9)	12 (8.0)		
Operation method				0.571	0.450
Open	566 (85.9)	440 (86.4)	126 (84.0)		
Closure	93 (14.1)	69 (13.6)	24 (16.0)		
Incision selection				3.738	0.253
Small	296 (44.9)	222 (43.6)	74 (49.3)		
S-shape	4 (0.6)	4 (0.8)	0 (0.0)		
L-shape	261 (39.6)	201 (39.5)	60 (40.0)		
八-shape	98 (14.9)	82 (16.1)	16 (10.7)		
Internal fixation				0.144	0.931
Screw	125 (19.0)	95 (18.7)	30 (20.0)		
Screw + Plate	420 (63.7)	326 (64.0)	94 (62.7)		
Screw + Plate + Bone graft	114 (17.3)	88 (17.3)	26 (17.3)		
Anesthesia				0.006	0.941
General anesthesia	362 (54.9)	280 (55.0)	82 (54.7)		
Local anesthesia	297 (45.1)	229 (45.0)	68 (45.3)		
Postoperative infection				1.062	0.303
Yes	22 (3.3)	15 (2.9)	7 (4.7)		
No	637 (96.7)	494 (97.1)	143 (95.3)		
Deep vein thrombosis of lower extremities				0.121	0.728
Yes	40 (6.1)	30 (5.9)	10 (6.7)		
No	619 (93.9)	479 (94.1)	140 (93.3)		
Reduction quality				0.210	0.646
Yes	497 (75.4)	386 (75.8)	111 (74.0)		
No	162 (24.6)	123 (24.2)	39 (26.0)		
Rehabilitation training				2.976	0.084
Yes	16 (2.4)	9 (1.8)	7 (4.7)		
No	643 (97.6)	500 (98.2)	143 (95.3)		
Weight-bearing time (months)				1.254	0.740
0–3	398 (60.4)	311 (61.1)	87 (58.0)		
4–6	210 (31.9)	161 (31.6)	49 (32.7)		
7–12	41 (6.2)	29 (5.7)	12 (8.0)		
> 12	10 (1.5)	8 (1.6)	2 (1.3)		

### Model variable screening

In the training cohort, gender, age, occupation, preoperative combined injuries, diabetes, waiting time for surgery, postoperative infection, deep vein thrombosis of lower extremities, and weight-bearing time were significantly associated with calcaneal fracture healing in univariate analysis (*P* < 0.20) (Table 2).

**Table 2:** Univariate analysis results related to postoperative recovery of calcaneal fracture [n (%)]

Variable	All	Good group	Poor group	*χ*^*2*^ value	*P* value
Gender				15.103	< 0.001*
Male	455 (89.4)	384 (91.9)	71 (78.0)		
Female	54 (10.6)	34 (8.1)	20 (22.0)		
Age (years)				21.219	0.001*
0–20	19 (3.7)	17 (4.1)	2 (2.2)		
21–30	58 (11.4)	53 (12.7)	5 (5.5)		
31–40	158 (31.0)	135 (32.3)	23 (25.3)		
41–50	133 (26.1)	113 (27.0)	20 (22.0)		
51–60	105 (20.6)	78 (18.7)	27 (29.7)		
> 60	36 (7.1)	22 (5.3)	14 (15.4)		
Ethnic origin				1.031	0.310
Han	488 (95.9)	403 (96.4)	85 (93.4)		
Others	21 (4.1)	15 (3.6)	6 (6.6)		
Occupation				17.092	0.004*
Student	16 (3.1)	14 (3.3)	2 (2.2)		
Office worker	41 (8.1)	33 (7.9)	8 (8.8)		
Farmer	229 (45.0)	186 (44.5)	43 (47.3)		
Manual worker	64 (12.6)	54 (12.9)	10 (11.0)		
Retired or Unemployed	20 (3.9)	10 (2.4)	10 (11.0)		
Others	139 (27.3)	121 (28.9)	18 (19.8)		
BMI (kg/m^2^)				1.859	0.602
< 18.5	14 (2.8)	13 (3.1)	1 (1.1)		
18.5–23.9	194 (38.1)	162 (38.8)	32 (35.2)		
24–27.9	223 (43.8)	181 (43.3)	42 (46.2)		
≥ 28.0	78 (15.3)	62 (14.8)	16 (17.6)		
Season				1.303	0.728
Spring	122 (24.0)	99 (23.7)	23 (25.3)		
Summer	167 (32.8)	134 (32.1)	33 (36.3)		
Autumn	162 (31.8)	135 (32.3)	27 (29.7)		
Winter	58 (11.4)	50 (12.0)	8 (8.8)		
Sanders classification				2.132	0.545
I	33 (6.5)	25 (6.0)	8 (8.8)		
II	198 (38.9)	162 (38.8)	36 (39.6)		
III	195 (38.3)	165 (39.5)	30 (33.0)		
IV	83 (16.3)	66 (15.8)	17 (18.7)		
Injury cause				3.212	0.351
Traffic accident	13 (2.6)	12 (2.9)	1 (1.1)		
Fall on the flat ground	11 (2.2)	8 (1.9)	3 (3.3)		
Fall from a high altitude	472 (92.7)	389 (93.1)	83 (91.2)		
Other	13 (2.6)	9 (2.2)	4 (4.4)		
Preoperative combined injuries				11.801	0.001*
Yes	168 (33.0)	124 (29.7)	44 (48.4)		
No	341 (67.0)	294 (70.3)	47 (51.6)		
Diabetes				2.631	0.105*
Yes	25 (4.9)	17 (4.1)	8 (8.8)		
No	484 (95.1)	401 (95.9)	83 (91.2)		
Hypertension				0.104	0.747
Yes	61 (12.0)	51 (12.2)	10 (11.0)		
No	448 (88.0)	367 (87.8)	81 (89.0)		
Coronary heart disease				0.453	0.501
Yes	19 (3.7)	14 (3.3)	5 (5.5)		
No	490 (96.3)	404 (96.7)	86 (94.5)		
Respiratory system disease				1.031	0.310
Yes	21 (4.1)	15 (3.6)	6 (6.6)		
No	488 (95.9)	403 (96.4)	85 (93.4)		
Other preoperative complications				0.082	0.775
Yes	43 (8.4)	36 (8.6)	7 (7.7)		
No	466 (91.6)	382 (91.4)	84 (92.3)		
Preoperative blister				0.002	0.962
Yes	33 (6.5)	27 (6.5)	6 (6.6)		
No	476 (93.5)	391 (93.5)	85 (93.4)		
Waiting time for surgery (days)				4.214	0.122*
0–7	318 (62.5)	269 (64.4)	49 (53.8)		
8–14	151 (29.7)	116 (27.8)	35 (38.5)		
> 14	40 (7.9)	33 (7.9)	7 (7.7)		
Operation method				0.050	0.822
Open	440 (86.4)	362 (86.6)	78 (85.7)		
Closure	69 (13.6)	56 (13.4)	13 (14.3)		
Incision selection				0.848	0.843
Small	222 (43.6)	183 (43.8)	39 (42.9)		
S-shape	4 (0.8)	4 (1.0)	0 (0.0)		
L-shape	201 (39.5)	166 (39.7)	35 (38.5)		
八-shape	82 (16.1)	65 (15.6)	17 (18.7)		
Internal fixation				0.195	0.907
Screw	95 (18.7)	79 (18.9)	16 (17.6)		
Screw + Plate	326 (64.0)	268 (64.1)	58 (63.7)		
Screw + Plate + Bone graft	88 (17.3)	71 (17.0)	17 (18.7)		
Anesthesia				0.048	0.827
General anesthesia	280 (55.0)	229 (54.8)	51 (56.0)		
Local anesthesia	229 (45.0)	189 (45.2)	40 (44.0)		
Postoperative infection				3.716	0.054*
Yes	15 (2.9)	9 (2.2)	6 (6.6)		
No	494 (97.1)	409 (97.8)	85 (93.4)		
Deep vein thrombosis of lower extremities				7.665	0.006*
Yes	30 (5.9)	19 (4.5)	11 (12.1)		
No	479 (94.1)	399 (95.5)	80 (87.9)		
Reduction quality				1.174	0.279
Yes	386 (75.8)	321 (76.8)	65 (71.4)		
No	123 (24.2)	97 (23.2)	26 (28.6)		
Rehabilitation training				0.612	0.434
Yes	9 (1.8)	6 (1.4)	3 (3.3)		
No	500 (98.2)	412 (98.6)	88 (96.7)		
Weight-bearing time (months)				33.904	< 0.001*
0–3	311 (61.1)	277 (66.3)	34 (37.4)		
4–6	161 (31.6)	119 (28.5)	42 (46.2)		
7–12	29 (5.7)	19 (4.5)	10 (11.0)		
> 12	8 (1.6)	3 (0.7)	5 (5.5)		

*P < 0.20

Multivariate logistic regression analysis showed that female (*OR* = 2.525, 95% *CI* 1.283–4.969), > 60 years (*OR* = 6.644, 95% *CI* 1.243–35.522), surgery within 8 to 14 days after fracture (*OR* = 2.172, 95% *CI* 1.259–3.745), postoperative infection (*OR* = 4.613, 95% *CI* 1.382–15.393), and weight-bearing time longer than 3 months after surgery (4–6 months, *OR* = 2.885, 95% *CI* 1.696–4.907; 7–12 months, *OR* = 3.030, 95% *CI* 1.212–7.578; > 12 months, *OR* = 15.589, 95% *CI* 3.244–74.912) were independent risk factors for calcaneal fracture recovery (Table 3).

**Table 3:** Multivariate logistic regression analysis results related to postoperative recovery of calcaneal fracture

	*B*	*S.E*	Wald *χ*^*2*^	*P*	*OR*	95% *CI*
Gender	0.926	0.345	7.195	0.007*	2.525	1.283–4.969
Age(years)			20.047	0.001*		
0–20	Ref					
21–30	− 0.213	0.923	0.053	0.817	0.808	0.132–4.931
31–40	0.504	0.816	0.382	0.537	1.656	0.334–8.202
41–50	0.463	0.818	0.320	0.572	1.589	0.320–7.897
51–60	1.301	0.813	2.559	0.110	3.672	0.746–18.072
> 60	1.894	0.855	4.902	0.027*	6.644	1.243–35.522
Waiting time for surgery(days)			8.085	0.018*		
0–7	Ref					
8–14	0.776	0.278	7.781	0.005*	2.172	1.259–3.745
> 14	0.029	0.483	0.004	0.952	1.030	0.400–2.654
Postoperative infection	1.529	0.615	6.182	0.013*	4.613	1.382–15.393
Weight-bearing time (months)			24.524	< 0.001*		
0–3	Ref					
4–6	1.060	0.271	15.289	< 0.001*	2.885	1.696–4.907
7–12	1.109	0.468	5.618	0.018*	3.030	1.212–7.578
> 12	2.747	0.801	11.760	0.001*	15.589	3.244–74.912

*P < 0.05

### Model validation and nomogram construction

**Fig. 3:**
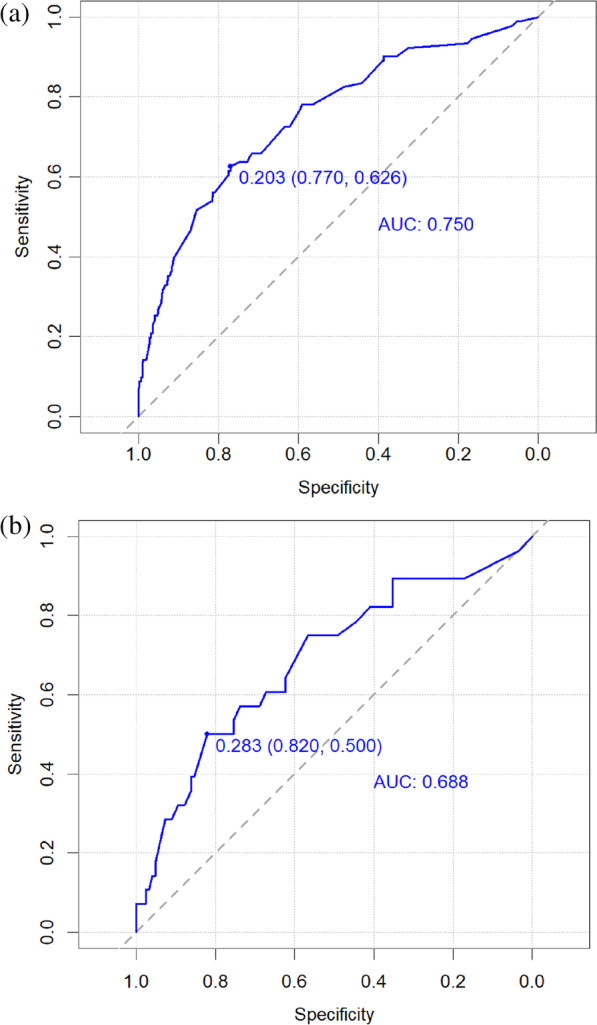
ROC curve of the prediction model for postoperative recovery of calcaneal fracture. **a** Training cohort, **b** Validation cohort

The C-indices were 0.750(95% *CI* 0.692–0.808) in the training cohort and 0.688(95% *CI* 0.571–0.804) in the validation cohort, illustrating that the model had a roughly medium level of discrimination. The internal validation also obtained a consistent conclusion, and the C-index was 0.715. ROC curves were constructed for both the training cohort and the validation cohort (Fig. 3).

**Fig. 4:**
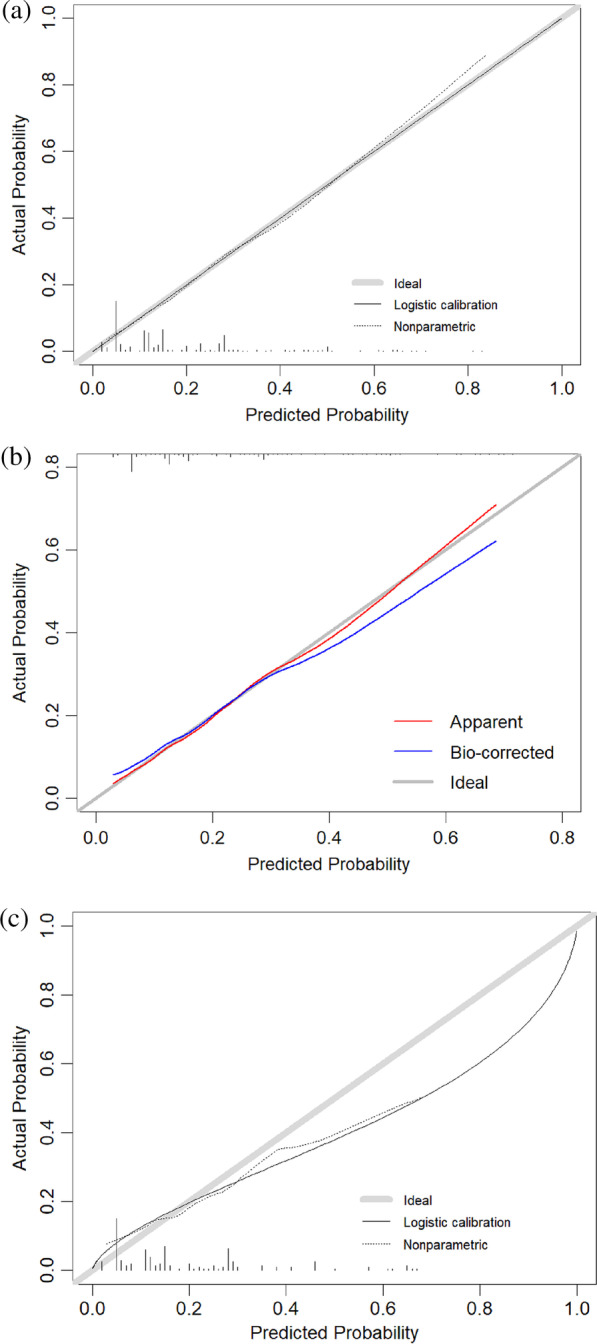
Calibration curve of the prediction model for postoperative recovery of calcaneal fracture. **a** Training cohort, **b**, Internal validation by bootstrap resampling **c** Validation cohort

On the calibration plots (Fig. 4), the model’s fitting curves were close to the ideal curves, indicating that model had considerable calibrating abilities. H–L test showed good fitting of the model, the* P* value was 0.87 in the training cohort and 0.50 in the validation cohort.

**Fig. 5:**
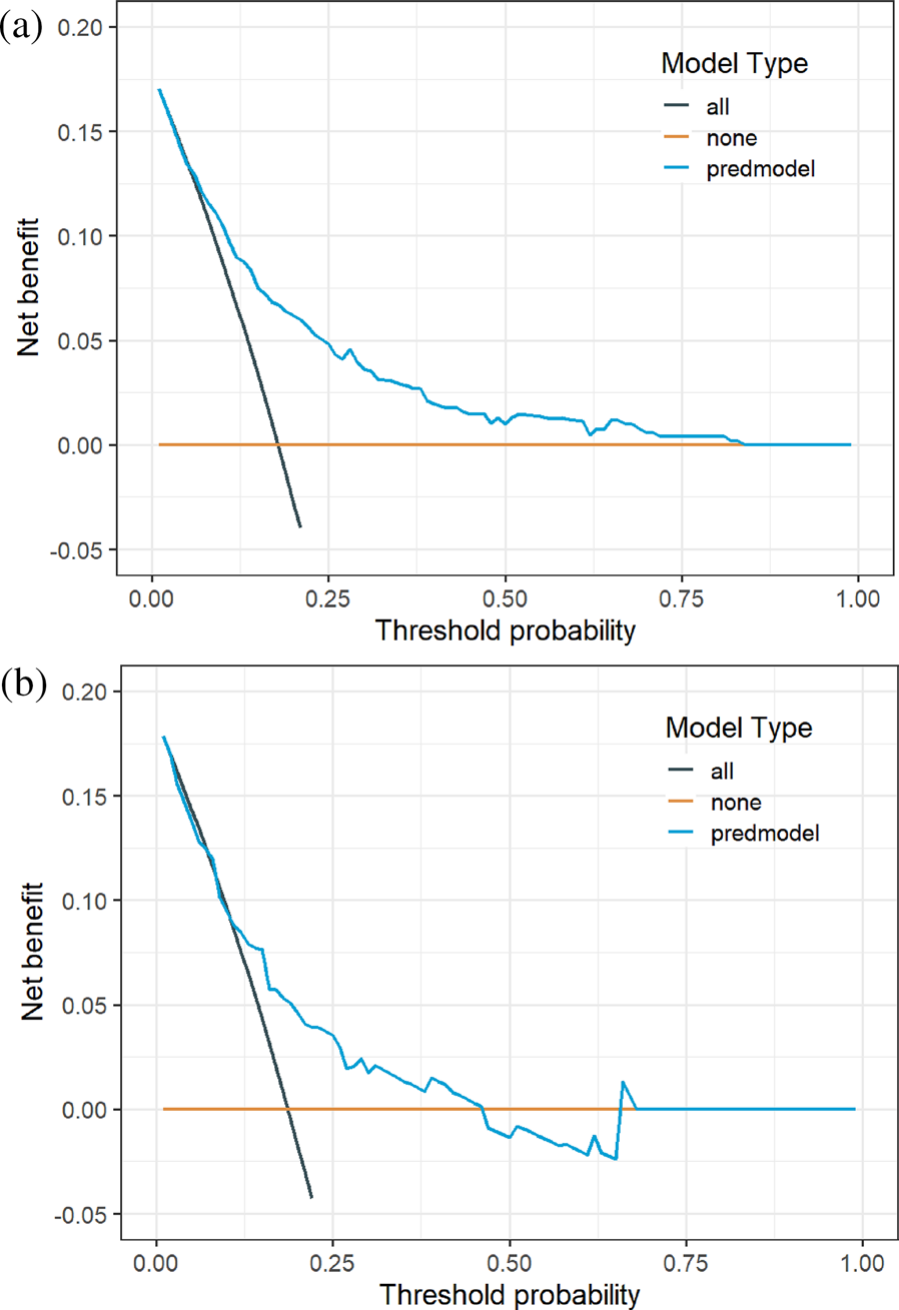
DCA curve of the prediction model for postoperative recovery of calcaneal fracture. **a** Training cohort, **b** Validation cohort

**Fig. 6:**
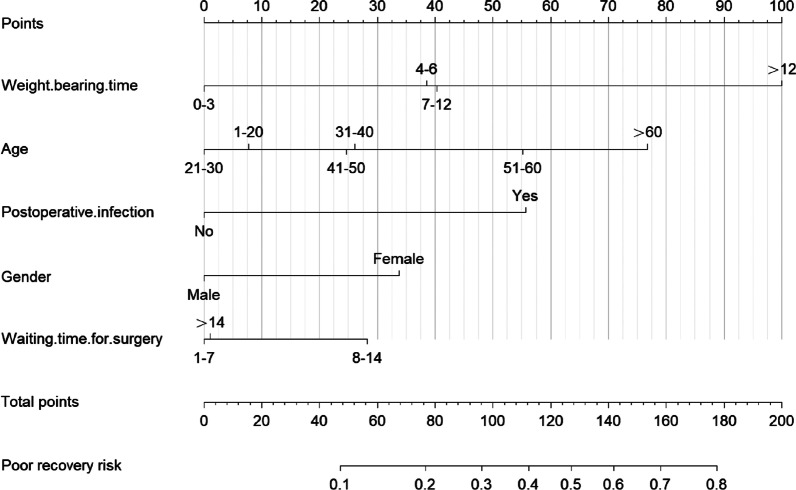
Nomogram of the prediction model for postoperative recovery of calcaneal fracture

**Fig. 7:**
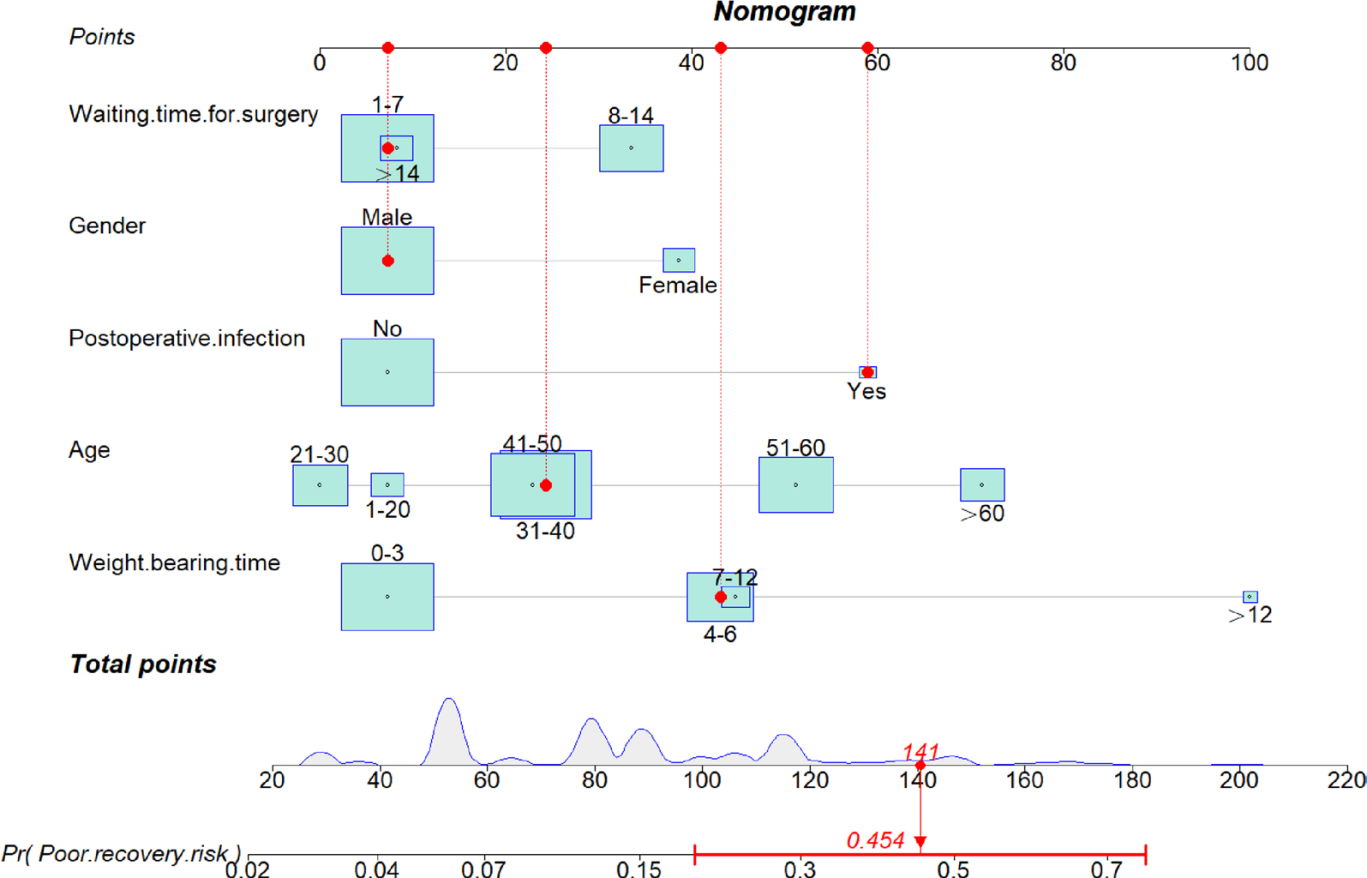
Schematic diagram of risk scoring on the nomogram

As shown in Fig. 5, according to the DCA curve, the best clinical effectiveness was achieved when the threshold probability was in the range of 0.10–0.45, and the net benefit of taking treatment measures was higher. A nomogram was used to visualize the results of the risk prediction model (Fig. 6). In practical application, the risk of poor postoperative recovery of calcaneal fractures can be determined based on the relevant variables of the individual. For example, for male patients with 31–40 years, postoperative infection, surgery within 7 days after fracture, and weight-bearing time within 4–6 months after surgery, the corresponding score was obtained on the nomogram according to the value of each factor. The risk of poor postoperative recovery was 0.454 (Fig. 7).

## Discussion

The calcaneus is the largest tarsal bone in the human body. Calcaneal fractures account for 60% of tarsal fractures, and intra-articular fractures account for the majority, up to 75% [20, 21]. And the displaced intra-articular calcaneal fractures are often accompanied by long-term sequelae, permanent disability and other adverse conditions [22]. Al-Mudhaffar et al*.* [23] reported that the incidence of wound complications of calcaneal fracture was 18.2% (6/33), 12.1% (4/33) of which were wound dehiscence, 3.0% (1/33) heel necrosis, and 3.0% (1/33) wound hematoma. And they all had wound infection (18.2%). 6.1% (2/33) of these patients subsequently developed deep infection and osteomyelitis. These seriously affected the postoperative recovery of patients, endangering their physical and mental health. Therefore, predicting the prognosis of calcaneal fractures can effectively improve the recovery effect and reduce pain in patients. In this study, a clinical predictive model was developed and validated to predict the risk of postoperative recovery in calcaneal fractures. According to the nomogram, weight-bearing time was the most important predictor, followed by age, postoperative infection, gender, and waiting time for surgery.

We found that weight-bearing exercise within 3 months after operation can improve the curative effect of fracture recovery. The postoperative recovery of patients who began to weight-bearing after 3 months was obviously poor, which may be due to non-weight-bearing conditions such as long-term bed rest or low-intensity walking, which slows down the process of local tissue from granulation tissue to callus formation to fracture healing [24]. This study concluded that early weight-bearing training was beneficial to the recovery of calcaneal function after operation. The result was consistent with the research results of some scholars [13, 25]. Therefore, we suggest that under the guidance of science, in the early stage of postoperative recovery of calcaneal fracture, weight-bearing rehabilitation exercises should be carried out according to the actual situation of patients, to promote functional recovery.

Female and older patients over 60 years were at higher risk for poor fracture recovery. Bone mass in the human body changes with age. Studies showed that men consistently gain bone mass at a faster rate than women [26]. Due to the decrease of estrogen in healthy women after menopause, osteoclast activity is enhanced, increasing bone turnover. At the same time, the decrease of estrogen level also leads to the decrease of parathyroid hormone secretion, resulting in lower blood calcium concentration and bone loss [27]. Therefore, the bone loss of elderly women is higher than that of men, resulting in poor recovery after fracture surgery. The bone mineral density appears physiological bone mass reduction with the increase of age [28], resulting in serious bone loss and calcium deficiency in the elderly, coupled with the metabolic physiological process delay, thus affecting fracture recovery. Choi et al*.* [29] showed that age was an independent risk factor for poor postoperative fracture healing.

In addition, our study found that postoperative infection also affected the postoperative recovery of calcaneal fracture. Incision infection can cause wound redness, swelling, exudation, and even skin flap necrosis. If not treated in time, this will further develop into calcaneal osteomyelitis, make calcaneus delayed union or malunion, and affect the postoperative curative effect of fracture [30]. At this stage, most scholars regard the postoperative complications as the outcome, and there are many studies on its influencing factors [31–33], but there is still a lack of articles exploring the impact on the postoperative curative effect as a related risk factor. Waiting time longer than 7 days for surgery after fracture was an independent risk factor for postoperative fracture healing. Su et al*.* [34] proved that longer than 14 days between injury and surgery could increase the risk of wound infection, thus affecting the postoperative effect, which was roughly similar to the study.

Consistent with the results of this study, in the study of Simske et al*.* [35], injury mechanism and fracture type were not related to the prognosis score. Similarly, Su et al*.* [34] found that preoperative injury, bone graft and diabetes did not affect postoperative recovery independently. However, some studies have found conflicting results. Wukich et al*.* [36] and Wu et al*.* [37] respectively concluded that diabetes and bone graft could increase the risk of poor postoperative outcome. Studies by Zhang et al*.* [38] and Schepers [39] have shown that high Sanders type is an independent risk factor for postoperative recovery. Nouraei et al*.* [40] showed that the proportion of patients with heavy manual work was significantly higher in patients who still needed arthrodesis after fracture surgery. In this study, we reached no conclusion regarding the curative effect of fracture recovery related to occupation, which may be due to differences in the population studied. The small incision for calcaneal surgery invented by Academician Zhang significantly reduces injury to the bone and soft tissue through accurate minimally invasive screw implantation. In our study, there was no statistically significant difference in postoperative recovery of calcaneal fractures treated with small incisions compared with other types of incisions (*P* > 0.05), which was consistent with the results of Wu et al*.* [41]. Consistent with this paper Abidi et al*.* [42] found that the surgical internal fixation of calcaneal fracture did not influence postoperative recovery. Preoperative blisters, non-compliance with reduction criteria and lack of systematic rehabilitation can slow down the postoperative recovery period of calcaneal fractures [43, 44]. There are few studies on the relationship between season, anesthesia, and the postoperative effect of calcaneal fracture, and the two factors were not correlated in our study.

The risk model can be used to quantitatively evaluate the postoperative recovery of calcaneal fracture and take individual control measures to assist clinical treatment. Our study has several limitations. Firstly, the retrospective design means that information bias is inevitable, and the classification of postoperative infection and pathogenic bacteria are not comprehensive enough. Second, as a single center study, the external malleability is poor, and the representativeness of the sample is low, which affects the accuracy of the results. This study needs a larger sample size, with a multicenter prospective approach to increase the scope of time and space to obtain data for a more comprehensive and accurate database.

## Conclusion

In summary, patients with female, > 60 years, surgery within 8–14 days after fracture, postoperative infection, and weight-bearing time longer than 3 months after surgery are at higher risk for poor postoperative recovery of closed calcaneal fracture. According to the risk prediction model, the postoperative prognosis of calcaneal fracture can be predicted, which can provide guidance for orthopaedic surgeons to make targeted preoperative examinations, surgical plans and rehabilitation training.

## Data Availability

All the data will be available upon motivated request to the corresponding author of the present paper.

## Abbreviations

BMI: Body mass index
ROC: Receiver operating characteristic
H–L: Hosmer–Lemeshow
DCA: Decision curve analysis
SD: Standard deviation
OR: Odds ratio
